# Do Not Lose Your Nerve, Be Callus: Insights Into Neural Regulation of Fracture Healing

**DOI:** 10.1007/s11914-023-00850-2

**Published:** 2024-01-31

**Authors:** Murad K. Nazzal, Ashlyn J. Morris, Reginald S. Parker, Fletcher A. White, Roman M. Natoli, Melissa A. Kacena, Jill C. Fehrenbacher

**Affiliations:** 1 Department of Orthopaedic Surgery, Indiana University School of Medicine, Indianapolis, IN USA; 2 Indiana Center for Musculoskeletal Health, Indiana University School of Medicine, Indianapolis, IN USA; 3 Department of Anesthesia, Indiana University School of Medicine, Indianapolis, IN USA; 4 Stark Neuroscience Research Institute, Indiana University School of Medicine, Indianapolis, IN USA; 5 Richard L. Roudebush VA Medical Center, Indianapolis, IN USA; 6 Department of Pharmacology and Toxicology, Indiana University School of Medicine, Indianapolis, IN USA

**Keywords:** Fracture, Peripheral nervous system, Central nervous system, Pain, AI, Artificial intelligence, ChatGPT

## Abstract

**Purpose of Review:**

Fractures are a prominent form of traumatic injury and shall continue to be for the foreseeable future. While the inflammatory response and the cells of the bone marrow microenvironment play significant roles in fracture healing, the nervous system is also an important player in regulating bone healing.

**Recent Findings:**

Considerable evidence demonstrates a role for nervous system regulation of fracture healing in a setting of traumatic injury to the brain. Although many of the impacts of the nervous system on fracture healing are positive, pain mediated by the nervous system can have detrimental effects on mobilization and quality of life.

**Summary:**

Understanding the role the nervous system plays in fracture healing is vital to understanding fracture healing as a whole and improving quality of life post-injury. This review article is part of a series of multiple manuscripts designed to determine the utility of using artificial intelligence for writing scientific reviews.

## Introduction

This is one of many articles evaluating the utility of using AI to write scientific review articles on musculoskeletal topics [1]. The first draft of this review was written entirely by humans. Refer to this edition’s Comment paper for more information [2]. Since human beings first walked the Earth, fractures have been among the most prevalent forms of traumatic injury. As it currently stands, fractures are the most common large-organ consequences of trauma [3]. Despite the plethora of research invested into developing preventative measures to reduce the incidence of trauma, fractured bones will persist. Furthermore, for a multitude of reasons, many demographics (e.g., aging population, type 2 diabetics, smokers, Alzheimer’s disease) are at increased risk of fracture complications, poor healing outcomes, and nonunion, which can cause enhanced and persistent pain at the fracture site [4]. As such, optimization of the healing process needs to be considered. Improving surgical techniques to expedite fracture healing, reduce infection risk, and reduce pain is one approach to optimize the healing process; however, increasing our understanding of fracture healing may also allow for the development of better pharmacological therapeutics for fracture healing and pain.

There are three fundamental stages to fracture healing: the reactive phase, the reparative phase, and the remodeling phase [5]. When examining the first two phases, the factors typically considered the most are angiogenic mediators, proinflammatory cytokines, and osteoprogenitor cells from the periosteum and bone marrow that undergo differentiation to produce new bone. Osteoprogenitor cells undergo endochondral ossification through differentiation into chondrocytes to establish a provisional cartilaginous matrix, which eventually develops into new trabecular bone that undergoes further remodeling to become cortical bone [6]. Beyond their role as critical components of fracture healing, these processes serve within homeostatic conditions to maintain a healthy bone microenvironment [7]. In the final phase, bone remodeling is mediated by osteoblasts (OBs), osteoclasts (OCs), and other cells directing ossification. Surprisingly, there is little discussion regarding the role of neural regulation in fracture healing at any of these stages; however, tangible evidence for a role of both the peripheral (PNS) and central nervous systems (CNS) to regulate fracture healing is evident. Furthermore, the nervous system is essential for the sensing and response to fracture pain. The objective of this review is to provide insight into the neural regulation of fracture healing and how the nervous system plays a role in mediating fracture pain.

## The Effects of the Peripheral Nervous System on Fracture Healing 

The PNS regulates many physiological attributes of the body’s non-nervous tissues and organs by relaying visceral and somatic information to the CNS. This function can be extended to apply to bone and fracture healing via two primary nerve types: sympathetic and sensory nerve fibers [8]. In addition to relaying information from the periphery to the CNS, peripheral nerves can also modulate peripheral tissues through the antidromic release of neurotransmitters and neuropeptides [9].

### Peripheral Nerve Reinnervation Following Fracture in Animal Models

Following traumatic injury, the body recruits a variety of cells and molecular factors which form the somatic inflammatory response. The recruited cells associated with fracture serve as first responders by releasing humoral cytokines which enhance the healing process. Nerve fiber recruitment is another significant component of the fracture healing process. Three days following rat tibial fracture, immunohistochemistry reveals the presence of growth-associated protein (GAP-43) in both the periosteum and the fracture hematoma [10], as an indicator of axonal growth and regeneration of free nerve endings within the organized hematoma. GAP-43 expression increases following fracture, so that at day 7 the nerve fibers are found penetrating the avascular cartilage of the callus and the hyperplastic periosteum [10]. Common to both sensory and sympathetic nerve populations is the dependence on neurotrophins to stimulate and maintain ingrowth of associated nerve fibers into bone tissue. The ingrowth of neuronal tissue prior to angiogenesis supports the concept of neurite outgrowth as a prerequisite for neovascularization, although this topic is still being discussed [11].

### Mixed Nerve Denervation in Animal Models

To investigate the fundamental role of peripheral innervation in fracture healing, sciatic nerve resection models have been used to effectively deplete the fracture site of nerve input [12–19]. Gross examination of tibial fractures with sciatic denervation has shown faster callus formation and union [13, 15, 16, 19]; however, denervated calluses have reduced overall RNA concentration during endochondral ossification, suggesting that there is compromised ability to generate bony matrix. Indeed, overall mineralization and woven bone density are reduced at the callus in denervated animals, having been replaced by bone marrow [15, 16]. Sciatic nerve resection as a means to investigate the role of the PNS on fracture healing is suboptimal: the efficacy of resection to induce complete denervation is questionable, as perivascular nerve growth and release of neuropeptides have been observed at the fracture site despite complete sciatic resection [15]. Furthermore, the sciatic nerve comprised of a mixture of efferent, afferent, and autonomic fibers; thus, these studies will not decipher the roles of specific nerve types. In fact, because the sciatic nerve carries motor fibers that innervate the entirety of the posterior thigh and leg, many of the findings may potentially be attributed to reduced loading and mobility, as opposed to actual reduced sensory or autonomic innervation of bone [20]. Indeed, tibial fractures without sciatic resection may cause bone loss in ipsilateral femurs, resulting in mechanically weaker bones [21]. Nonetheless, while these points should certainly be considered, the sciatic nerve denervation models still provide some insight into the general effects of peripheral nerves during fracture healing.

Comparing fracture healing in animals with intact versus resected sciatic nerves is not the only way the functional role of the sciatic nerve has been examined. Low-intensity pulsed ultrasound (LIPUS) is reported to accelerate fracture healing. The ultrasound waves propagate mechanical energy absorbed by the fracture environment, resulting in cellular responses that improve healing [19]. To elucidate the mechanisms of LIPUS, one study examined how effects of LIPUS on fracture healing change with sciatic denervation [19]. Positive effects of LIPUS are attenuated significantly following sciatic nerve resection, suggesting that LIPUS enhances fracture healing through activation of the peripheral nerves in the fracture microenvironment [19]. Overall, sciatic nerve innervation seems to propagate fracture healing, although more selective models of peripheral nerve manipulation are needed.

### Sensory Nerve Effects on Healing

In addition to indiscriminately modulating peripheral nerves by resecting or globally stimulating the nerves, it is possible to study specific nerve fiber types via activation by fiber-type specific electrical stimulation or through pharmacological or genetic inactivation of specific nerve fibers. Sensory nerves have been isolated as one of the two primary fiber types that innervate bone, so special examination of their function in fracture healing is critical. Some studies have examined bone formation with sensory electrical stimulation of the dorsal root ganglia (DRG), using rat models of non-fracture and fracture bone growth assessment [22, 23••]. In an attempt to elicit bony bridging between L4 and L5 transverse processes, investigators electrically stimulated L4 to L6 DRG (lower lumbar region), in the absence of bone decortications or bone grafting. Novel bony bridging was observed in all rats examined at L4/L5 transverse processes and most rats at L5/L6 transverse processes, with no fusion or bridging in rats without electrical stimulation [22]. In osteoporotic rats with a closed femoral fracture, electrical stimulation of L3 and L4 DRGs resulted in fractures that healed with greater bone mineral density (BMD) and mechanical stability [23••], supporting the notion that sensory fiber activation is osteogenic. A similar role of sensory neurons was established when examining fracture healing in the presence of sensory neuron inactivation, elicited by local capsaicin injections. Sensory denervation resulted in acute reductions of collagen I fiber upregulation 3 days post-fracture, and collagen II expression 1 week post-fracture was also impaired [24]. Collagens I and II are both necessary components of fracture healing and bone homeostasis, playing roles in endochondral ossification and forming much of the organic bone matrix [25]. Just as the sciatic nerve resections resulted in larger callus formation in several instances, but with reduced ossification and generally weaker bone, sensory nerve denervation resulted in a similar trend where biomechanical testing demonstrated that denervated fractures bore ~ 21% less force to failure compared to innervated fractures [24]. This pro-healing role for sensory neurons is not altogether surprising, as many neuropeptides critical for fracture healing are primarily secreted by sensory neurons, including calcitonin gene-related peptide (CGRP) and substance P (SP), and their release is upregulated almost immediately following fracture in humans [26].

### Autonomic Regulation of Fracture Healing

Autonomic nerve fibers also play a role in fracture healing. The primary focus of autonomic research has largely been to examine a role for the sympathetic neurons, despite the expression of receptors for neurotransmitters released by both sympathetic and parasympathetic nerve fibers within the cells of the bone marrow microenvironment [27]. Systemic ablation of sympathetic fibers, through peripheral 6-hydroxydopamine (6-OHDA) injections, has been used to induce sympathectomies [28]. Loss of sympathetic innervation has been shown to reduce trabecular bone volume fraction (BV/TV) and mechanical strength of both fractured and unfractured bones [28, 29]. There are also differences in callus maturation timelines with sympathectomy. Dividing callus maturation into three phases—mesenchymal, cartilaginous, and bony—studies found that sympathectomy delayed callus maturation at multiple timepoints after fracture [30, 31]. In addition, fractured and undamaged bones from animals with sympathectomy have weaker bone biomechanics, as they are significantly less resistant to torque and have reduced stiffness. BV/TV, connectivity density, trabecular bone thickness, and separation are all adversely affected with sympathectomy in fractured and unfractured bone [31]. The adverse effects of sympathectomy can be restored with local repletion of vasoactive intestinal peptide or with systemic injections of a β3 adrenergic agonist [29], suggesting that multiple neurotransmitters could be responsible for the positive effects of sympathetic nerves on bone healing and quality. Changes in immune cells in the fracture callus were also changed with sympathectomy, as CD4 + and CD8 + cells were significantly reduced both early on (5 days) and later (3 weeks) in the fracture healing process, suggesting that an interaction between sympathetic nerves and the immune system could also underlie the effects of sympathetic nerve loss on fracture healing. Because of the possible interactions between systemic sympathetic nerve ablation and the immune system, the effects of limiting sympathetic nerve denervation to the lower trunk or fracture site on fracture healing were examined. Surgical procedures to remove parts of the sympathetic trunk are methods that have been used in several instances and can be performed at different segmental levels (cervical, lumbar, periarterial). In lumbosacral ganglionectomies performed in the early twentieth century on patients, the subsequent loss of sympathetic innervation resulted in increased blood flow to the lower extremities [32, 33]. Furthermore, this form of sympathectomy resulted in increased bone growth in paralyzed patients with poliomyelitis in the lower extremity. In animal models, cervical sympathetic trunk resection elicited an increase in BMD, BV/TV, and trabecular bone 1–2 weeks after mandibular fracture in a model of distraction osteogenesis (DO) [34, 35]. It was subsequently determined that sympathectomy diminished the levels of norepinephrine (NE) and its corresponding receptor, β3-adrenergic receptor (adrb3), on mesenchymal stem cells (MSCs) at the site of distraction. Subsequent in vitro studies established that osteoanabolic factors in MSCs, including alkaline phosphatase (ALP), runt-related transcription factor 2 (RUNX2), and osteocalcin (OCN), were reduced upon exposure to NE, and that these effects were antagonized by deletion of the β3-adrenergic receptor [35]. NE is not the only neurotransmitter being used by sympathetic nerves to guide bone homeostasis and healing. Some sympathetic nerves are postnatally induced by interleukin-6 (IL-6) to switch to a cholinergic-releasing phenotype [36]. When this subset of ACh-releasing nerves is ablated, there is a decrease in bone mass. Furthermore, increases in bone mass through exercise appear to be mediated through a concurrent increase in the number of cholinergic sympathetics innervating the bone [36].

Altogether, peripheral nerve denervation studies have demonstrated that ablation has various effects on fracture healing, depending upon the breadth of denervation. Systemic sensory and sympathetic nerve and focal cholinergic sympathetic nerve loss generally diminish bone mineralization, whereas focal ablation of noradrenergic sympathetic neurons promotes healing and mineralization. Although some mechanistic factors were discussed above, the next section will expand on how the peripheral nerves affect bone homeostasis and fracture healing.

## Molecular Factors in Peripheral Regulation

As evidenced by the studies described above, many nerve ablation models have conflicting results and interpretations of data. However, understanding neurogenic effects within the microenvironment of the fracture site can expand our understanding of fracture healing and provide opportunities for pharmacological therapeutics that accelerate bone repair. Several neuropeptides have already been recognized as osteoanabolic [37], and examinations of CGRP, SP, vasoactive intestinal peptide (VIP), and neuropeptide Y (NPY) are already popular targets. However, recent studies describing a role for these neuropeptides in fracture healing will be described further. Refer to Table 1 for a summary of factors in the PNS involved in fracture healing.

**Table 1:** Peripheral nerve-related molecular factors in bone. Factors listed have been shown to promote bone formation and/or improve fracture healing. Most factors promote osteogenic expression, although VIP has shown thus far to only inhibit OCs

Molecular factor	Primary receptor(s)	Function in bone
CGRP	CLR and RAMP1 [38]	↑ osteogenic gene expression [38]
SP	NK-1R [39]	↑ osteocalcin and collagen expression [39, 40]
VIP	VPAC1 and VPAC2 [41, 42]	↓ OC differentiation [41, 42]
NPY	Y1 [43]	↑ RUNX2 expression in MSCs [43]
BDNF*	TrkB [44]	↑ OPG expression [44]
NGF*	TrkA [20, 45]	↑ BMP and VEGF expression [46, 47]

*Not made endogenously by peripheral nerves

### Calcitonin Gene-Related Peptide

CGRP is a polypeptide primarily known for its nociceptive signaling as a neurotransmitter and as a vasodilatory, proangiogenic molecule [38]. Its main receptors are the calcitonin gene-related peptide receptor (CLR) and receptor activity modifying protein 1 (RAMP1). In addition to its known principal functions, CGRP can potentially bind to OBs, as OBs express CLR. This results in increased osteogenic gene expression of factors such as osteoprotegrin (OPG) [38]. The expression of CLR on OBs in conjunction with proangiogenic properties of CGRP makes it a prime candidate for improved bone healing. Cell culture studies support this notion as well. In cell culture studies, transfected M2 macrophages were manipulated to overexpress CGRP in vitro. At 1 and 3 days following transfection, CGRP impaired mRNA expression of pro-osteogenic genes bone morphogenetic proteins-2 (BMP-2) and -6 (BMP-6), wnt10, and oncostatin M within the M2 macrophages, but then pro-osteogenic gene expression was increased at 5 and 7 days post-transfection [48]. The investigators then co-cultured transfected M2 macrophages with MC3T3 osteoblastic precursor cells and assessed osteoblastic differentiation and osteogenic gene expression. Expression of osteoblastic differentiation mRNA factors ALP, RUNX2, osterix, and osteopontin in MC3T3 cells when co-cultured with the CGRP-overexpressing M2 macrophages decreased and then increased in a time-dependent fashion that mirrored osteogenic gene expression in the M2 macrophages. All these gene expression effects were negated by the use of veterporfin, a yes-associated protein-1 (Yap-1) inhibitor, suggesting that the osteogenic effects of CGRP are mediated by Yap-1. Another study found that pharmacological inhibition of CGRP signaling results in reduced phosphorylated Ras/extracellular signal-regulated kinase (pERK) in the fracture microenvironment [49]. Activated ERK has been shown to promote OB differentiation; thus, altered pERK activity could be another mechanism by which CGRP promotes fracture healing. Further, CGRP increases OB production of cyclic adenosine monophosphate (cAMP), increases IGF-1 production, and inhibits the proinflammatory cytokine tumor necrosis factor-α (TNF-α), a promoter of osteoclastogenesis [50, 51].

In animal models, while CGRP-deficient mice have shown no changes in fracture healing in one instance [30], other studies show impaired callus formation, maturity, and OB activity in fracture healing [49, 52, 53•]. Research to specifically alter CGRP signaling at the site of fracture or in defined cell populations has helped to understand some of the discrepancies within knockout animals. A prominent role for CGRP in altering proliferation and activation of osteoblasts has been suggested. One recently defined role for CGRP is to drive the proliferation of periosteal progenitor cells following fracture. In these experiments, CGRPexpressing nerve fibers were shown to innervate the periosteum and expression of CGRP receptor components on periosteal progenitor cells was identified. To interrogate the role of the CGRP, investigators depleted the CGRP receptor on the periosteal progenitor cells, resulting in a loss of callus size and extent of cartilage in the callus [53•]. Surprisingly, however, there was little effect of these manipulations on biomechanical properties of the fractured bones. Another study looked at the effects of CGRP-impregnated fibrin sealant to determine whether it would affect patellar regeneration following partial patellectomy. In these experiments, CGRP sealant increased bone area and BMD compared to control, whereas inclusion of the CGRP antagonist in the fibrin sealant trended towards decreasing bone area and BMD compared to control at 8 and 16 weeks post-operatively in rabbits [54]. Moreover, patella-patellar tendon complexes possessed greater load to failure and stiffness than controls and antagonists. As mentioned previously, acute increases in CGRP release via electrical stimulation of the DRG improve fracture healing in osteoporotic fractures in rats [23••], an effect that was reversed by administration of a CGRP antagonist. Overall, these increases in CGRP with fracture and the pro-osteogenic signaling mechanisms of the neuropeptide suggest that it promotes fracture healing.

### Substance P

The primary receptor of SP is the neurokinin-1-tachykinin receptor (NK-1R). Antagonism of this receptor reduced the expression of osteocalcin and collagens I and II [40]. Moreover, biomechanical testing demonstrated that NK-1R inhibition reduced the loading capacity of the femurs both acutely (6 weeks post-operatively) and chronically (3 months post-operatively). Impairment of SP might produce these results due to its activation of the wnt signaling pathway, as fracture experiments in type 1 diabetes (T1D) rats demonstrate evidence of compromised wnt signaling. SP treatment of these rats restored wnt signaling, increased OPG, reduced RANKL expression, and improved fracture healing [39]. Thus, at least in T1D rats, SP induces osteogenesis.

### Vasoactive Intestinal Peptide

Although the neuropeptide VIP is typically thought of in the context of the intestinal tract as a promoter of digestion, VIP receptor types 1 (VPAC1) and 2 (VPAC2) are expressed on OCs, prevent OC differentiation, and have been examined as a potential therapeutic in inflammatory bone disease [41, 42]. Furthermore, it has recently been the focus of examination in the context of fracture healing. Not surprisingly, VIP expression is significantly reduced with sympathectomy [28]. Exogenous VIP treatment to animals symphathectomized by 6-OHDA treatment partially restored bone volume losses and biomechanical deficits induced by the sympathectomy, suggesting a pro-osteogenic role for VIP in fracture healing [28].

### Neuropeptide Y

A role for NPY, which is expressed primarily in noradrenergic sympathetic neurons, in fracture healing has also been examined. Increased expression of NPY can lead to osteogenesis of MSCs by binding to Y1 receptors, the primary NPY receptor, and upregulating RUNX2 expression [43]. Deletion of the Y1 receptor results in delayed healing; while wild-type mice experience partial to complete bridging 6 weeks after fracture, Y1-deficient mice did not experience any cases of complete bridging 6 weeks post-surgery [55]. An examination of NPY-secreting nerve fiber distribution in angulated fractures found that NPY fibers penetrate the periosteum and fibrous callus, especially on the concave side of an angular fracture, and innervate the tissue on that side [56]. Because the concave side of an angulated fracture is associated with bone formation, it is thought that NPY innervation is involved, in part, in this process during fracture healing. As observed with CGRP inhibition, NPY inhibitors reduce phosphorylated ERK in fractures [49]. Altogether, NPY release appears to be pro-osteogenic, and loss of NPY signaling may be responsible for the loss of fracture healing observed with sympathectomy in animal fracture models.

### Neurotrophins

Neuropeptides are not the only factors involved in the healing process. Brain-derived neurotrophic factor (BDNF) is a neurotrophin that has been shown to accelerate fracture healing [57]. BDNF treatment enhances the proliferation of MLO-Y4 osteocyte-like cells in vitro and the differentiation of MSC into OBs [58]. BDNF works through binding to its receptor, tropomyosin-related kinase B receptor (TrkB), resulting in activation of the Akt signaling pathway and subsequent inhibition of asparagine endopeptidase (AEP). Genetic knockout of AEP results in increased trabecular bone density and can partially reverse the loss of bone density induced by ovariectomy [44]. Moreover, the use of a TrkB receptor agonist, R13, produced equivalent results and increased OPG expression in the bone. This specific study also found that 7,8-DHF, a BDNF agonist, inhibits RANKL and promotes OPG as well. Interestingly, another study found essentially the opposite results when examining 7,8-DHF in fracture healing. In their study, 7,8-DHF treatment reduced callus sizes and the mechanical stability of healed bones, and had no effects on pro-OB RNA expression [59]; however, these studies were performed in male mice. Clearly, more work needs to be done to fully understand the effects of BDNF on fracture healing and whether there is a sex-specific effect.

Another neurotrophin, nerve growth factor (NGF), can potentially play a significant role in healing. Its receptor, tyrosine kinase receptor type 1 (TrkA), is widely expressed in osteoprogenitor cells and OBs, and NGF itself has been shown to be produced by OBs [20, 45]. NGF has been shown to upregulate vascular endothelial growth factor (VEGF) and BMP expression in bone [46, 47]. Expectedly, it is thus deemed an accelerator and promoter of fracture healing [37, 60•]. Injections of β-NGF post-fracture in mice promote endochondral ossification and result in union up to 2 weeks faster compared to mice with no treatment [6]; thus, NGF improves overall outcomes and at a faster rate than in the absence of exogenous NGF treatment.

## The Effects of Central Nervous System Injury on Fracture Healing

Superficially, it may appear that the CNS involvement in fracture healing is sparse, but there are significant interactions between the CNS and bone. Direct pathways linking the CNS to bone healing have flourished in the past two decades, with much research centered on the use of traumatic injury models.

### Traumatic Brain Injury

The literature regarding the effects of traumatic brain injury (TBI) on fracture healing is conflicting. Systemic inflammation caused by traumatic injury to the head often results in increased proinflammatory cytokine circulation, which can stimulate bone resorption and reduce bone formation [61, 62]. Central influences of these injury-related responses include diminished release of pituitary hormones such as growth hormone (GH) secretion, which can alter aspects of osteogenesis [62–64]. From a neurological perspective, hyperadrenergic activity leading to autonomic dysfunction after acute brain injury is not uncommon following TBI [65]. An increased sympathetic state effectively suppresses osteoblastogenesis and promotes osteoclastogenesis through direct targeting of β adrenergic receptors found on OBs, resulting in bone loss [66].

### Clinical Findings

Despite the potentially negative effects of TBI on fracture healing, many in the orthopedic community perceive nervous system trauma to be an accelerant of fracture healing. Numerous reports have been published describing faster fracture healing outcomes in patients, due, in part to robust callus formation, in conjunction with the coincidence of TBI and fracture [67–71]. A multitude of subsequent clinical and animal studies have been conducted to further validate or reject these findings. Some of these studies have found that patients with head injury exhibit a time to fracture union and healing that is significantly reduced when compared to those with no head injury [67, 72, 73, 74•]. This phenomenon has been reproduced in fracture injury of femurs, tibiae, and humeri [67, 72, 73]. Moreover, the callus-to-femoral diaphysis ratio is greater in head injury patients as assessed by radiographs [72, 73, 74•, 75]. Patients with TBI concomitant to fracture can have greater callus volume than fracture-only counterparts [76]. When these phenomena were investigated on a cellular level, it has been shown that serum of patients with TBI can result in higher proliferation rates of human fetal osteoblast (hFOB) cells and expression of OB mRNA differentiation markers [73]. Further in vitro analysis of hFOB1.19 and primary OB response to TBI patient serum collected up to 1-week post-injury finds significant increases in their proliferative capacity [75]. Contradictory findings have also been observed when examining the correlation between severity of TBI and healing in patients; whereas a strong positive correlation between the extent of TBI and callus ratio exists in one study [74•], another study found no correlation [77]. Histologic examination of the “callus,” at 3 weeks post-injury, shows mature woven bone in the periarticular periphery indicative of potential heterotopic ossification as opposed to normal fracture healing responses [67].

### Preclinical Findings

Discrepancies regarding the relationship between TBI and fracture healing also exist in rodent models. In rats, callus diameter is significantly reduced 3 weeks post-injury as opposed to normal fractures, but the opposite results are observed in mice [78–80]. Despite this indicating improved outcomes in mice, one study found mice subjected to repeated mild TBI had significant reductions in mineralized bone formation at the callus site, as well as reduced bone connectivity density [81]. Nonetheless, there is a majority consensus of improved healing. Assays of callus mineral density and increased torsional strength show modest increases in mice 4 weeks post-operatively in a severe TBI plus fracture model [80]. Callus stiffness, an indicator of healing, in head-injury rats is greater than bones in non-TBI rats, again supporting that TBI hastens the fracture healing process [78]. Moreover, C3H10T_1/2_ cell line proliferation is increased in vitro when exposed to serum derived from TBI rats, indicating increased MSC proliferation [78], although this serum had no growth effects on NIH3T3 fibroblast cell line or OB lineage cells.

### Underlying Mechanisms

Just as the effects of CNS injury on bone healing are complex, the underlying molecular basis behind these effects is poorly defined. It is evident there are multiple mechanisms that regulate healthy bone, and it is likely that neurological, hormonal, and humoral factors all play a role in directing healing outcomes. When delving into the subject from a neurological perspective, some key neuropeptides and neurotransmitters which have been described previously to promote fracture healing come into play. Refer to Table 2 for a summary of molecular factors involved with CNS injury and where they are located. One factor is NPY, which has been found to be elevated in TBI patient groups with fracture [43]. Following TBI, there is an increase of NPY in the cerebrospinal fluid (CSF). This increase extends to the serum due to leakage of CSF that often results because of CNS injury.

**Table 2:** Molecular factors increased following CNS injury. These factors have all been shown to improve fracture healing. However, where they are found to increase following TBI differs

Molecular factor	Reported site of increase
CGRP	DRG and serum [51, 82–85]
NPY	CSF [43]
NGF	Serum [84, 86]
EGF	Serum [84, 86]

Another factor is CGRP, which plays a role in both the PNS and CNS. Following TBI with fracture, there is an increase of CGRP concentrations in the DRG [82]. Most studies report an increase in CGRP in the serum following CNS trauma [51, 83, 84], yet this is not entirely consistent [82]. Similarly, NGF and epidermal growth factor (EGF) are neurotrophins [85] that are elevated in serum following TBI and fracture, suggesting that they might mediate TBI-induced improvements in fracture healing [84, 86].

## Neural Connections to Fracture Pain

Much of the discussion thus far has examined the effects of neural function within the bone microenvironment. With some exceptions, the nervous system works to accelerate fracture healing. However, sensory nerve activation also mediates fracture pain. While bone repair should be the primary goal for the treatment of fracture patients, doing so in a way that can also diminish the pain associated with trauma and bone fracture is critical. Indeed, alleviating acute pain generally has positive effects on bone healing [87]. Between 30 and 50% of fracture patients develop chronic pain following bone fracture [88, 89]; thus, understanding the changes in the nervous system that are induced by fracture is critical to address both acute and chronic fracture pain.

In the periosteum, there is a diffuse presence of sensory Aδ and C fibers, which are subtypes that are predominantly nociceptive in nature. These fibers, upon fracture, become damaged and send nociceptive signals to the brain [90]. Increases in NGF and other inflammatory mediators in the bone microenvironment result in enhanced peripheral sensitization [91]. Examination of interventions that may attenuate pain by decreasing inflammation and nociceptive signaling while maintaining healing has been highlighted in recent animal studies. Administration of anti-NGF therapy in mice has been shown to reduce pain behaviors up to 70% while maintaining bone healing outcomes [92, 93]. However, anti-NGF therapy has also been labeled as having a multitude of adverse effects, making it a suboptimal candidate for widespread therapy. Interestingly, one recent finding has shown that exogenous delivery of adenosine can mitigate the nociceptive effects of NGF and reduce pain while simultaneously improving healing outcomes in mice [94]. In addition to NGF, caspase-6 has been identified as a potential prime regulator of fracture pain, and its inhibition in mice has also shown reduced pain behaviors following fracture [95]. These recent findings may provide a potential therapeutic mechanism for improved fracture healing outcomes with reduced pain in the future.

During the healing process, nerve fibers begin to sprout into the healing bone, as early as 3 days post-fracture [10]. Normal healing dictates that this is subsequently followed by synaptic pruning to avoid impingement and overstimulation. However, ectopic nerve sprouting may result in impaired pruning and constant stimulation. Aberrant signaling by peripheral sensory neurons, either via peripheral sensitization or via continuous activation of impinged axons, can drive the development of central sensitization, whereby structural, functional, and chemical changes in the CNS amplify peripheral input to enhance the perception of pain. One functional change observed with fracture in the nervous system is an increase in glial cell proliferation. One to 4 weeks following fracture, there is increased expression of astrocyte and microglial gene markers in the peripheral and central somatosensory systems [96]. In the spinal cord, increased microglia were found to be associated with allodynia, reduced weight bearing, and heat hypersensitivity, as treatment with a microglial inhibitor reduced nociceptive responses. Moreover, systemic inhibition of SP through the injection of a neurokinin 1 (NK1) receptor antagonist reduced microglia and astrocyte activation and subsequent increases in nociceptive hypersensitivity in a mouse fracture model [96], suggesting a role for afferent neuropeptide signaling in driving activation of microglia and astrocytes.

Nonunion is another significant driving force of pain development [97, 98]. Nonunions in mice have been shown to present a significantly increased pain response, accompanied by CGRP and GAP-43-positive increased nerve density in the bone marrow [97, 99]. Indeed, it is suggested that ongoing pain in nonunion may be a result of improper pruning and the development of neuroma-like nerve endings within the bone [99]. Furthermore, in nonunion studies in rodents, there is an increased expression of proinflammatory cytokines TNF-α and interleukin-1β (IL-1β) in the serum [100], potentially leading to increased pain at the fracture site.

Not all chronic pain develops due to nonunion. Complex regional pain syndrome (CRPS) is a pathologic condition in which the body responds abnormally to tissue injury, resulting in chronic pain [101, 102••]. While NGF overexpression plays a role in developing this condition, humoral response of increased proinflammatory cytokines is among the chief underlying causes of CRPS [98]. The release of these cytokines, however, is still neuronally mediated, with CGRP and SP increases likely being the driving force for this cytokine storm [101, 103]. Chemically sympathectomized mice have a significantly higher paw withdrawal threshold and increased weight-bearing 3 and 7 weeks post-fracture than those with fracture only [102••], suggesting that sympathetic nerves drive some of the pain in CRPS. The opposite was true for parasympathetic nerves, as administration of nicotine, as a parasympathetic agonist, inhibited pain behaviors in the CRPS animal model. Both sympathectomy and nicotine significantly decreased IL-1β release [102••], suggesting that the mechanism of action is via modulation of the immune response to injury. Although the nervous system’s presence at the fracture site is crucial for proper healing, it can also propagate the development of pain.

## Conclusion

**Fig. 1:**
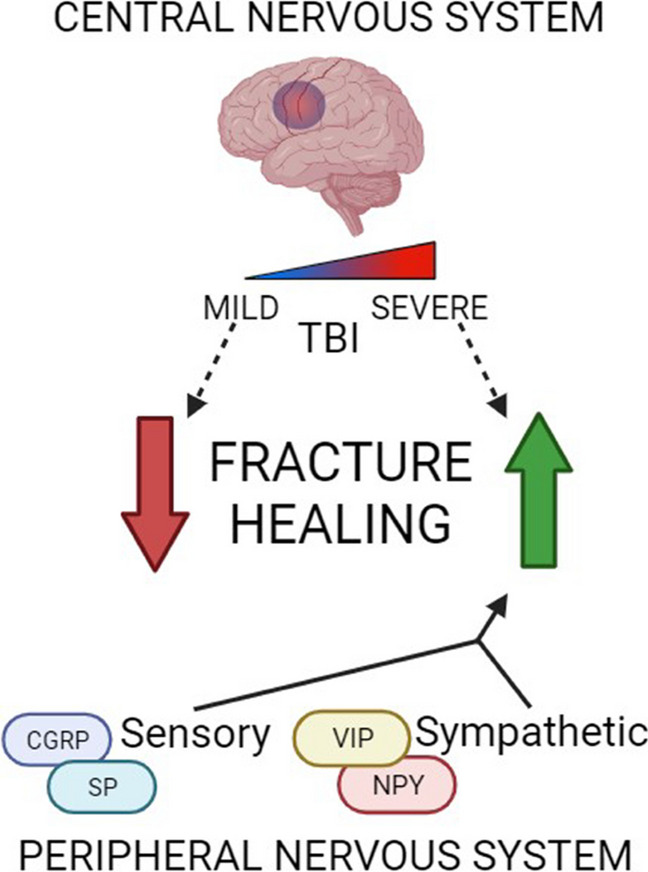
Nervous system regulation of fracture healing. The effects of TBI on fracture healing are contested; however, evidence suggests that mild brain injury impairs healing, whereas severe brain injury promotes healing. Peripheral nerves innervating the fracture site secrete a variety of factors that promote healing. Created with BioRender.com

The nervous system is important in the fracture healing process. In the PNS, increased stimulation of the fracture with sensory nerve fibers proves critical in creating a more mechanically stable, healed bone. On the other hand, although a diffuse sympathetic response is necessary, focal sympathetic innervation of the fracture appears to have dual effects: adrenergic sympathetic nerves slow healing, while cholinergic sympathetic nerves accelerate healing. Many of the effects of the PNS are mediated through secreted molecular factors, which unfortunately also activate nociceptors in accordance with evolutionary protective measures to guard against injury. Thus, improved healing outcomes with neural regulation largely coexist with a caveat of increased pain. Traumatic injuries to the brain result in altered peripheral neurotrophin and neuropeptide release and some poorly understood changes in fracture healing outcomes and timeline. These effects, as well as the effects of the PNS on fracture healing, can be observed in Fig. 1. Given the uncertainty of the roles of the PNS and CNS in fracture healing, there is continuing need for increased research into neural regulation of fracture healing. Understanding how the PNS and CNS regulate fracture healing and how they contribute to the development of acute and chronic pain is critical for future exploration of therapeutic interventions that could target the pain pathways while minimizing negative effects on the fracture healing process.
